# Microbial interactions within the plant holobiont

**DOI:** 10.1186/s40168-018-0445-0

**Published:** 2018-03-27

**Authors:** M. Amine Hassani, Paloma Durán, Stéphane Hacquard

**Affiliations:** 1 Department of Plant Microbe Interactions, Max Planck Institute for Plant Breeding Research, 50829 Cologne, Germany; 2 Environmental Genomics, Christian-Albrechts University of Kiel, 24118 Kiel, Germany; 3 Max Planck Institute for Evolutionary Biology, 24306 Plön, Germany

**Keywords:** Microbe-microbe interactions, Holobiont, Plant microbiota, Competition, Cooperation

## Abstract

Since the colonization of land by ancestral plant lineages 450 million years ago, plants and their associated microbes have been interacting with each other, forming an assemblage of species that is often referred to as a “holobiont.” Selective pressure acting on holobiont components has likely shaped plant-associated microbial communities and selected for host-adapted microorganisms that impact plant fitness. However, the high microbial densities detected on plant tissues, together with the fast generation time of microbes and their more ancient origin compared to their host, suggest that microbe-microbe interactions are also important selective forces sculpting complex microbial assemblages in the phyllosphere, rhizosphere, and plant endosphere compartments. Reductionist approaches conducted under laboratory conditions have been critical to decipher the strategies used by specific microbes to cooperate and compete within or outside plant tissues. Nonetheless, our understanding of these microbial interactions in shaping more complex plant-associated microbial communities, along with their relevance for host health in a more natural context, remains sparse. Using examples obtained from reductionist and community-level approaches, we discuss the fundamental role of microbe-microbe interactions (prokaryotes and micro-eukaryotes) for microbial community structure and plant health. We provide a conceptual framework illustrating that interactions among microbiota members are critical for the establishment and the maintenance of host-microbial homeostasis.

## Background

**Fig. 1:**
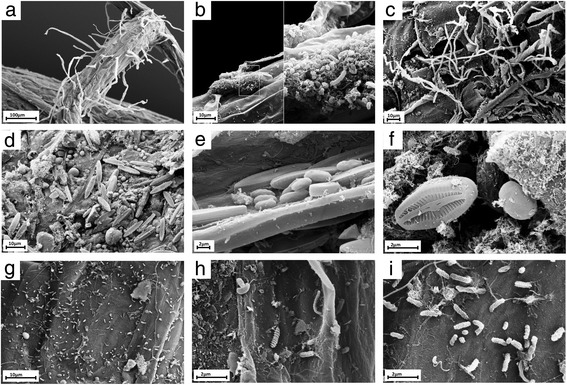
Microbial consortia naturally formed on the roots of *Arabidopsis thaliana*. Scanning electron microscopy pictures of root surfaces from natural *A. thaliana* populations showing the complex microbial networks formed on roots. **a** Overview of an *A. thaliana* root (primary root) with numerous root hairs. **b** Biofilm-forming bacteria. **c** Fungal or oomycete hyphae surrounding the root surface. **d** Primary root densely covered by spores and protists. **e**, **f** Protists, most likely belonging to the *Bacillariophyceae* class. **g** Bacteria and bacterial filaments. **h**, **i** Different bacterial individuals showing great varieties of shapes and morphological features

In nature, healthy and asymptomatic plants cohabit with diverse microbes such as archaea, bacteria, fungi, and protists (collectively termed the plant microbiota, see an example for *Arabidopsis thaliana* root microbiota in Fig. 1) that form complex microbial consortia and impact plant growth and productivity [1–4]. Although plants have evolved their own adaptations to alleviate most biotic and abiotic stresses in nature, they also rely on their microbial partners to survive and defend themselves against microbial invaders [5]. Several studies have reported a wide range of beneficial effects of microbiota members on plant health including disease suppression [6, 7], priming of the plant immune system [8], induction of systemic resistance [9], increased nutrient acquisition [10], increased tolerance to abiotic stresses [11], adaptation to environmental variations [12], or promotion of the establishment of mycorrhizal associations [13]. Interactions between plants and their associated microbial communities are not unidirectional. The host plant also provides novel metabolic capabilities to its microbial associates, leading to the adaptation of niche-specialized inhabitants that can either have positive (mutualistic), neutral (commensalistic), or deleterious (pathogenic) impact on plant fitness [14].

A current concept considers the multicellular host and its associated microbiota as a functional entity called the “holobiont,” in which evolutionary selection likely occurs between the host and the microbes but also among microbes [15]. Although extensive evidence supports the co-evolution of binary interactions between plants and pathogenic or symbiotic microbes, it remains unclear whether genomic signatures exist for the microbial community-related fitness phenotypes in the host genome and its associated microbiome. It is likely that not all microorganisms within the plant holobiont affect each other’s evolution trajectories and that selective pressure does not only impact holobiont fitness in a positive way [16–18]. Although the evolution of plant-microorganism partnership and particularly of mutualism has been discussed in respect of the holobiont concept [18], the evolution of microbe-microbe interaction mechanisms that favor co-existence of highly diverse microbial consortia on or inside plant habitats remains poorly described. A more comprehensive understanding of the evolution of microbe-microbe-host interactions remains challenging due to the complex ecological interactions taking place in nature and the different ways plant-associated microbes are inherited (i.e., vertical transmission via seeds [19–21] vs. horizontal acquisition from the environment [22, 23].

**Fig. 2:**
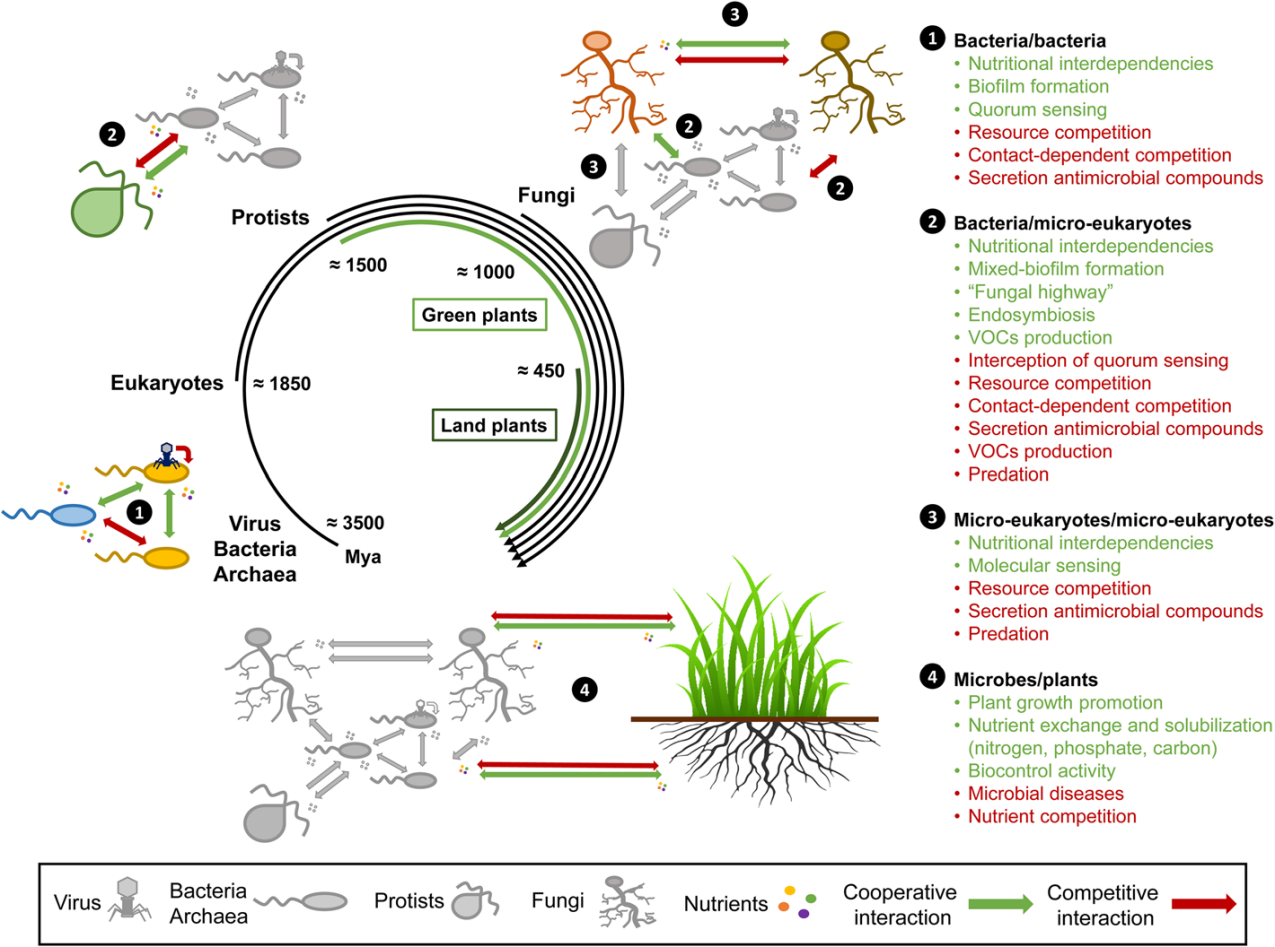
Evolutionary history of microbe-microbe and plant-microbe interactions. Microbial interactions are outlined at the evolutionary scale, showing that plant-microbe interactions occurred relatively recently compared to the more ancestral interactions among bacteria or between different microbial kingdoms. Both competitive (red) and cooperative (green) interactions within and between microbial kingdoms are depicted. Mya, million years ago. Evolutionary divergence estimated from [237, 238]

The very ancient origin of microbes on Earth, tracing back to the beginning of life more than 3.5 billion years ago, indicates that microbe-microbe interactions have continuously evolved and diversified over time, long before plants started to colonize land 450 million years ago (Fig. 2). Therefore, it is likely that both intra- and inter-kingdom intermicrobial interactions represent strong drivers of the establishment of plant-associated microbial consortia at the soil-root interface. Nonetheless, it remains unclear to what extent these interactions in the rhizosphere/phyllosphere and in endophytic plant compartments (i.e., within the host) shape microbial assemblages in nature and whether microbial adaptation to plant habitats drive habitat-specific microbe-microbe interaction strategies that impact plant fitness. Furthermore, the contribution of competitive and cooperative microbe-microbe interactions to the overall community structure remains difficult to evaluate in nature due to the strong environmental noise. To mitigate these technical hurdles, reductionist approaches have been primarily used to identify several of the diverse and sophisticated molecular mechanisms used by microbes to cooperate and compete on plant tissues and persist as complex microbial consortia [24–27].

We focus this review on three microbial groups (bacteria, fungi and protists) that are abundantly found on plant tissues and briefly describe the diverse mechanisms used by these microbes to cooperate and compete *in planta*. We further discuss how these microbial interactions represent important organizational rules structuring the plant microbiota as well as their impact on plant growth and health.

## Composition of plant-associated microbial communities and structuring factors

### The bacterial and archaeal microbiota of plants

It is now widely accepted that bacterial community establishment on plants is not random but rather controlled by specific assembly rules [4, 22, 23]. The factors impacting the structure of bacterial communities in plants include soil type [28, 29], plant compartment [30–34], host genotype/species [29, 35–42], plant immune system [24, 43], plant trait variation/developmental stage [43–47], and residence time/season [48–53]. Despite the large number of bacterial phyla described in nature and the multiple factors that affect these communities, the bacterial microbiota of plants is dominated by three major phyla (Proteobacteria, Actinobacteria, and Bacteroidetes) in both above- and belowground plant tissues [22, 54]. Extensive overlap between root- and leaf-associated community members has been described at OTU (operational taxonomic unit) level resolution in grapevine, agave, wild mustard, and *A. thaliana* [30, 31, 34, 55, 56], and the reciprocal relocation between root- and leaf-associated bacterial communities has been further validated using microbiota reconstitution experiments with germ-free *A. thaliana* [31]. Despite the striking structural similarities observed between *A. thaliana* leaf- and root-associated bacterial communities, large-scale genome sequencing and re-colonization of germ-free plants revealed that host-associated microbiota members are specialized and adapted to their respective cognate plant organs [31]. Although Archaea represent abundant members of the plant microbiota (i.e., 35.8%) [57], they have been less studied than their bacterial counterparts, most likely because no pathogenic isolates have been described to date. Plant endophytic archaeal taxa primarily belong to the phyla Thaumarchaeota, Crenarchaeota, and Euryarchaeota, but their functional relevance for their plant host remains to be clarified [57].

### The fungal microbiota of plants

Even though less attention has been given to the fungal microbiota of plants, culture-independent community profiling revealed a staggering diversity of fungi colonizing both above- and belowground plant tissues, mainly belonging to two major phyla: Ascomycota and Basidiomycota [58–60]. In roots, although arbuscular- (Glomeromycota phylum) and ectomycorrhizal fungi have been mostly studied, recent community profiling data indicate that other endophytic fungi also represent an important fraction of the fungal root microbiota [59, 61]. In non-mycorrhizal plant species such as *A. thaliana*, *Arabis alpina*, or *Microthlaspi*, it has been proposed that they might compensate for the lack of mycorrhizal partners [62–64]. Similar to bacteria, the structure of plant-associated fungal communities is not random [65–67] and varies depending on soil type, plant compartment, plant species, or seasons [55, 68–72]. Compared to the bacterial microbiota, fungal communities established in soil and on plant roots seem to be more subjected to stochastic variations [73–75] and respond differently to environmental factors [76–78]. Consequently, mainly dispersal limitation and climate explain the global biogeographic distribution of fungi and have been suggested to constrain fungal dispersal, favoring high endemism in fungal populations [79–81]. Consistent with that, simultaneous investigation of both fungal and bacterial communities associated with plants suggested a greater importance of biogeography for structuring fungal communities compared to bacterial communities [55, 61, 71, 82]. Whether this pattern is accentuated by the different taxonomic resolutions resulting from 16S rRNA and ITS marker loci remains to be clarified [83].

### Plant-associated protists: the neglected fraction of the plant microbiota

Most of the protists that are known to interact with plants belong to the Stramenopiles-Alveolata-Rhizaria supergroup (SAR) [84], and particularly those belonging to the Oomycota (Stramenopiles) and Cercozoa (Rhizaria) lineages. Within Oomycota, few members belonging to the genera *Pythium*, *Phytophthora*, *Peronospora* (and other downy mildew genera), or *Albugo* are often found living in association with plant roots or leaves [85–88]. Notably, the vast majority of oomycete species described so far are highly destructive plant pathogens that have a major impact on plant productivity worldwide [89]. Nonetheless, root colonization by closely related oomycetes (*Pythium* spp.) can have contrasted effect on plant growth, and specific strains (i.e., *Pythium oligandrum*) were reported to confer fitness benefit to the host [90–92], suggesting that some members can establish non-pathogenic interactions with plants. Although profiling of oomycetal communities in healthy plant tissues remains sparse, recent reports indicate a very low diversity [88, 93], with members of the *Pythiaceae* family being the most represented on plant tissues [93]. Within Cercozoa, one of the dominant protistan groups in terrestrial ecosystems, community profiling data revealed an unexpectedly high diversity in plant roots and leaves [93, 94], as well as a strong plant filtering effect on community structure, pointing to specific niche adaptation to plant habitats. Taken together, these data support the importance of considering protists, and particularly Oomycota and Cercozoa members for holobiont fitness.

## Cooperative interactions among plant microbiota members

Although the structure of microbial communities formed in the leaves and roots of several plant species has been elucidated, there is still a lack of knowledge regarding how cooperation between plant-associated microbiota members influences microbial community establishment and plant health. In the following paragraphs, we summarize the cooperative mechanisms that are employed by microbiota members to persist within the plant holobiont (Fig. 2).

### Nutritional interdependencies

These interactions refer to the reciprocal exchange of metabolites between nutritionally dependent microbes to compensate metabolic deficiencies [95–97]. Using this strategy, microbes can extend their fundamental niches to persist in nutrient-poor environments [98, 99], access recalcitrant compounds that cannot be easily broken down, remove toxic metabolites, or exchange electrons [95, 96]. Such metabolic exchanges appear widespread among microbes, including soil, water, and the human gut bacteria [100]. For example, the rhizobacterium, *Bacillus cereus UW85*, tightly associates with and stimulates the growth of bacteria from the Cytophaga-Flavobacterium group (CF, Bacteroidetes) in the soybean rhizosphere. The growth-promoting mechanism likely involves bacterial cell wall components, since peptidoglycan isolated from *B. cereus* cultures stimulated the growth of the CF rhizosphere bacterium *Flavobacterium johnsoniae* in vitro [101]. Nutritional interdependencies likely promote beneficial interactions and increase connectedness among community members, which might ultimately result in adaptive gene loss [96, 102]. Evolution of nutritional dependencies through gene loss is well described for endosymbiotic bacteria inhabiting hyphae of mycorrhizal or soil-borne fungi [103] but remains to be more thoroughly investigated among plant microbiota members. Evolution of dependencies through gene loss might occur between microbiota members if partner fidelity is high between microbes colonizing plant tissues and if a redundant function can be complemented by the presence of the other. In this case, independency can be irreversibly lost without any gain of function. Determining whether plant microbiota members display a low or high degree of metabolic complementarity will not only have critical implications for microbiome research and synthetic microbial ecology but also provide novel insights into how evolutionary processes act on holobiont components.

### Biofilm formation

Biofilms are micro-architectural constructions that embed microbial communities. The secretion of extracellular polymeric substances to build biofilms requires microbial cooperation [104, 105]. These secretions, which are the result of combined processes from clonal or multispecies microbial consortia [106, 107], provide selective advantage for microbes such as protection from competitors and antimicrobial molecules [108], activation of enzymatic processes that require high cellular density [109], and acquisition of new genes via horizontal gene transfer [110]. Recently, it has been shown that biofilm-mediated microcolonies formed on root hairs of finger millet by a root-inhabiting bacterial endophyte (*Enterobacter* sp.) confer a physical and chemical barrier that prevents root colonization by the pathogen *Fusarium graminearum* [111]. Importantly, bacterial traits related to motility, attachment, and biofilm formation are needed for the anti-*Fusarium* activity *in planta*. These results suggest that a complex interplay takes place between the bacterium and root-hair cells, leading to the formation of this specialized killing microhabitat [111]. Although the formation of biofilm has been mainly described for plant-associated bacteria (Fig. 1) [112, 113], mixed bacterial-fungal biofilms or bacterial biofilm formed on the surface of fungal hyphae seems common on plant tissues [26, 114]. Recently, it has been shown that bacterial ability to form a biofilm on fungal hyphae is widely shared among soil bacteria but rarely occurs on the hyphae of ascomycete fungi. Notably, the ability of *Pseudomonas fluorescens BBc6* to form a biofilm on the hyphae of the ectomycorrhizal fungus *Laccaría bicolor* is enhanced at the vicinity of the ectomycorrhizal root tip, suggesting that the establishment of the ectomycorrhizal symbiosis stimulates bacterial biofilm formation on fungal host surfaces [115]. Taken together, these studies indicate that biofilm formation on plant tissues represents a hotspot for microbial interactions that locally shape microbial assemblages.

### Molecular communications

These mechanisms correspond to stimuli and responses used by microbes to sense other microbes, activate specific biological processes, and/or gauge population density. One of the most described mechanisms is known as quorum sensing, which is used by several Gram-negative bacteria to monitor their own population densities through the production of the signaling molecule *N*-acyl-*l*-homoserine lactone (AHL) [116]. AHLs have a conserved chemical structure, varying in length and nature at the C3 position of the acyl chain. Consequently, different bacterial taxa can produce the same signal molecule type and cooperate or interfere (quorum quenching) with other unrelated taxa. This crosstalk phenomenon is supported by the fact that 8 to 12% of isolates from rhizobacterial libraries can activate AHL-specific reporter strains (biosensor) in vitro. The authors’ results suggest that AHLs serve as a universal language for bacteria-bacteria communication in the rhizosphere [117]. Importantly, quorum sensing is also likely important for inter-kingdom communication between bacteria and plant-associated fungi, as recently shown in the animal field [118]. Quorum sensing signal production and regulation have also been evidenced in the case of certain fungi such as *Saccharomyces cerevisiae* and *Candida albicans*, an opportunistic human fungal pathogen*.* The latter secretes the signaling molecule farnesol to control filamentation [119, 120], to inhibit biofilm formation [121], and to activate oxidative stress responses or drug efflux [122, 123]. Similar quorum sensing mechanisms have not been yet thoroughly described for plant-associated fungi. Nevertheless, and beyond quorum sensing mechanisms, numerous microbial compounds such as volatile organic compounds (VOCs), oxalic acid, trehalose, glucose or thiamine have been reported to act as signaling molecules, triggering directed movement between rhizospheric bacteria and fungi and promoting fungal-bacterial associations [124–126]. It is therefore tempting to speculate that the sensing mechanisms used by soil- and plant-associated microbes are highly diverse and evolutionarily ancient, arising long before plant-microbe associations occurred (Fig. 2).

### Enhanced dispersal

Although motile bacteria can independently move by using different mechanisms (e.g. swimming, swarming, etc.), they remain dependent on other microbes to efficiently disperse, especially in water-unsaturated soils [127]. Although this phenomenon has been primarily described for interactions between bacteria and filamentous eukaryotes, it is likely that other root-associated micro-eukaryotes such as motile protists can also serve as a carrier for fungal spores or bacterial cells along the soil-root continuum. It has been well demonstrated that specific bacteria can use hyphae of filamentous eukaryotes as a vector, the so-called “fungal highway,” giving them a selective advantage at spreading in their environments [128, 129]. Particularly, motile bacteria use fungal mycelium’s hydrophobicity to reach faster and solubilize pollutants, which opens a promising branch of research for bioremediation purposes [128, 130, 131]. These mycelial networks have been shown to facilitate horizontal gene transfer between spatially separated bacteria, by providing continuous liquid films in which bacterial migration and contacts are favored [132]. Consistent with the tight physical association observed between plant-associated fungi/oomycetes and bacteria [114], it is also likely that specific microbiota members use fungal and oomycetes hyphae and mycelial networks as vectors to colonize the plant endosphere (i.e., within plant tissues) [133]. This hypothesis is consistent with the fact that bacterial communities associated with the roots of *Bistorta vivipara* plants are spatially structured up to a distance of 60 cm, whereas no spatial structure was observed for soil bacterial communities [134]. According to a recent report on community assembly in cheese rind microbiota [135], it is also likely that fungal networks established along the soil root continuum may also favor the growth of motile over non-motile bacteria at the root vicinity.

### Bacterial endosymbiosis in fungi

This cooperative mechanism includes the highly specialized interaction that occurs between plant-associated fungi and their bacterial endosymbionts [136]. The bacteria, detected in the fungal cytoplasm, can be actively acquired from the environment [137] and, in most cases, vertically inherited via fungal spores [138, 139]. Several examples of bacterial endosymbionts that live in intimate association with plant-associated fungi (i.e., *Rhizophagus*, *Gigaspora*, *Laccaria*, *Mortierella*, *Ustilago*, *Rhizopus* sp.) have been reported and mostly belong to the families *Burkholderiaceae* or related [138, 140–142], *Bacillaceae* [143, 144], or are Mollicutes-related endobacteria [145]. Such interactions can impact the reproductive fitness of both members; for example, the bacterial endosymbiont (*Burkholderia* sp.) of a pathogenic *Rhizopus* fungus produces a toxin that provides fitness benefit to the fungus and is required for successful fungal colonization of rice plants [140]. This bacterium is also required for fungal reproduction, and its absence impairs fungal spore formation [139]. Interestingly, spores of the arbuscular mycorrhizal fungus *Gigaspora margarita* can host both *Burkholderia*- and *Mollicutes*-related endobacteria, supporting the idea that some root-associated fungi have their own intracellular bacterial low-diversity microbiome [146]. Taken together, these data suggest that fungal-bacterial symbioses are widespread and may influence the outcome of plant-fungal associations.

## Competitive interactions among plant microbiota members

Plant-associated microbiota members also engage in direct or indirect competition with closely or distantly related-microbiota members. These competitive mechanisms are diverse and likely have cascading consequences on microbial community structure and stability, as well as on host-microbiota homeostasis. In the following paragraph, we describe the competitive mechanisms employed by plant microbiota members for successful niche colonization (Fig. 2).

### Resource competition

Microbes can use indirect mechanisms to compete with other microbes, such as rapid and efficient utilization of limiting resources. For instance, bacteria have evolved sophisticated strategies to sequestrate iron via secretion of siderophores, subsequently altering the growth of opponent microbes in their immediate vicinity [147–149]. Nutrient sequestration is also recognized as an important trait of biocontrol agents to out-compete pathogens [25, 150]. For example, the secretion of iron-chelating molecules by beneficial *Pseudomonas* spp. has been linked to the suppression of diseases caused by fungal pathogens [151]. Furthermore, it has been recently shown that resource competition is an important factor linking bacterial community composition and pathogen invasion in the rhizosphere of tomato plants [152]. These results not only underline the role of resource competition for microbial interactions, but also indicate their relevance for plant health.

### Contact-dependent competition

Plant-associated bacteria can engage in direct antagonistic interactions mediated by contact-dependent killing mechanisms. These are largely mediated by the bacterial type VI secretion system, a molecular weapon deployed by some bacteria (mostly Proteobacteria) to deliver effectors/toxins into both eukaryotic and prokaryotic cells [153]. The plant pathogen *Agrobacterium tumefaciens* uses a puncturing type VI secretion system to deliver DNase effectors upon contact with a bacterial competitor in vitro and in the leaves of *Nicotiana benthamiana*. Remarkably, this contact-dependent antagonism provides a fitness advantage for the bacterium only *in planta*, underlining its specific importance for niche colonization [154]*.* In addition, the essential role of the bacterial type III secretion system for bacterial-fungal and bacterial-oomycetal interactions has been illustrated several times in the literature, suggesting that bacteria employ this strategy to successfully colonize a broad range of eukaryotic hosts (plants, animals, small eukaryotes) [155–158]. For instance, it has been reported that *Burkholderia rhizoxinica* utilizes this secretion system apparatus to control the efficiency of its symbiosis with the fungal host, *Rhizopus microsporus*, and that mutants defective in such secretion system display a lower intracellular survival and fail to provoke fungal sporulation [156]. Contact-dependent competitive mechanisms seem widespread among bacteria and are likely relevant for both intra- and inter-kingdom microbe-microbe interactions.

### Secretion of antimicrobial compounds

Numerous plant-associated microbes have been shown to secrete chemical compounds that directly suppress the growth of microbial opponents [159]. Filamentous eukaryotes are well known to produce a multitude of low-molecular-weight secondary metabolites that have antifungal activities against phylogenetically unrelated microbes (such as acetylgliotoxin and hyalodendrin) [160, 161]. These secondary metabolites are often silent in pure culture and only activated in co-culture or in a community context [162–165], indicating their specific role in competitive interactions. Bacteria also produce different metabolites, including antibiotics and enzymes that exhibit broad-spectrum activity against phylogenetically unrelated fungal plant pathogens [166, 167]. Antagonistic interactions among bacteria have been reported to be important in the structuring of soil-, coral-, or plant-associated bacterial communities [168–170]. Notably, the study of antagonistic interactions among bacterial isolates from the rhizosphere, the roots, and the phyllosphere of the medicinal plant *Echinacea purpurea* suggests that plant-associated bacteria compete against each other through the secretion of antimicrobials [170]. Moreover, bacteria from different plant compartments showed different levels of sensitivity to antagonistic activity, thereby indicating that antagonistic interactions might play an important role in shaping the structure of the plant microbiota [170].

### Emission of volatile organic compounds

In addition to antibiotic production, different bacteria (*Pseudomonas*, *Serratia*, *Stenotrophomonas*, *Streptomyces*) can also produce VOCs that act as infochemicals within and between microbial groups and have been shown to inhibit the growth of a broad diversity of plant-associated fungi and oomycetes [171–173]. Recently, it has been shown that bacterial VOCs also drive species-specific bacteria-protist interactions and likely serve as signals for protists to sense suitable prey. Notably, a *Collimonas pratensis* mutant, defective in terpene production, lost the ability to affect protists activity, indicating that terpenes represent key components of VOC-mediated communication between bacteria and protists [174]. Although the VOC activity of fungi/oomycetes towards bacteria has been less investigated, recent data indicate that soil filamentous microbes can also produce volatile blends that are perceived by bacteria. Schmidt and colleagues identified over 300 VOCs from soil and rhizospheric fungi/oomycetes and demonstrated that some can be sensed by bacteria, thereby influencing their motility [126]. Soil bacteria have also been shown to produce VOCs (reviewed in [171]). The best illustrated example is the genus *Streptomyces* that is known to produce sesquiterpenes exhibiting antimicrobial activity [172]. More recently, the comparative genomic analysis of the six strains of *Collimonas* have revealed that *C. pratensis* harbor functional terpene synthase genes responsible for the biosynthesis of a blend of sesquiterpenes with antimicrobial properties [173]. Taken together, these results suggest that VOCs produced by bacterial and fungal members of the plant microbiota act as an additional defense line against other microbes and are also likely important for long distance structuring of the microbial communities [171].

### Predation

As well-known among macroorganisms, microbes can also predate on other microbes at the root-soil interface. For instance, bacterial mycophagy consists on bacteria’s ability to actively grow at the expense of living fungal hyphae [175, 176]. Recently, it has been suggested that diverse mycophagous bacteria colonize saprotrophic rhizosphere fungi and feed as secondary consumers on root-derived carbon [177]. Similarly, specific fungi can grow, feed, and reproduce on other fungi (i.e., mycoparasitism), leading to the death of the latter [178]. This lifestyle appears to be ancient since it has been dated to at least 400 million years ago based on fossil records [179]. Some fungal or oomycetal species belonging to the genus *Trichoderma* or *Pythium*, respectively, can parasite or antagonize other fungi or oomycetes and can be used as biocontrol agents for plant protection, since they can also intimately interact with plant roots without causing disease symptoms [92, 180, 181]. Root-associated bacteria can also prey on other bacteria as described for *Bdellovibrio* spp. Phylogenetic and prey range analyses suggested that root-associated *Bdellovibrio* spp. differ from those in the soil, likely because these bacteria are best adapted to prey on root-associated bacteria [182]. Protist predation on bacteria has been also well documented [183], and recent microbiota reconstitution experiments in microcosm indicate a clear effect of Cercomonads (Rhizaria: Cercozoa) grazing on the structure and the function of the leaf microbiota [184]. Their results indicate that Alpha- and Betaproteobacteria are less resistant to grazing and that predation restructures the bacterial network in leaves and influences bacterial metabolic core functions [184]. These data are consistent with the hypothesis that microbes are trophic analogs of animals and that trophic networks are likely important organizational rules for microbiota establishment [185].

## Importance of intermicrobial interactions for structuring plant-associated microbial communities

**Fig. 3:**
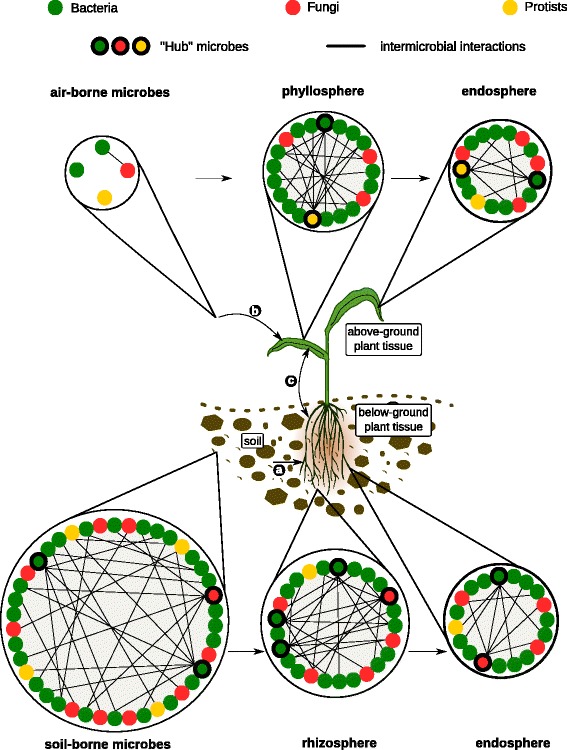
Representative microbial networks in different plant habitats. The figure illustrates microbial communities in the soil, air, rhizosphere, phyllosphere, and inside plant tissue (endosphere). In each of these habitats, microbes (represented by colored circles) could interact positively, negatively, or do not interact with other microbes (no lines). Specific microbes, often defined as “hub” or “keystone” species (circles highlighted in bold), are highly connected to other microbes within the networks and likely exert a stronger influence on the structure of microbial communities. (a) Root-associated microbes mainly derive from the soil biome. (b) Leaf-associated microbes originate from various sources such as aerosols, insects, or dust. (c) Relocation between aboveground and belowground microbiota members. The combination of microbe-microbe and host-microbe interactions is proposed to be critical for the establishment of the plant microbiota

The various mechanisms employed by microbes to cooperate and compete on plant tissues suggest that microbe-microbe interactions play fundamental roles in shaping and structuring microbial networks in nature. Therefore, the combination of host-microbe and microbe-microbe interactions is likely critical for the establishment of complex and diverse multi-kingdom plant-associated microbiota (Fig. 3) [186, 187]. However, the mechanistic understanding of the intermicrobial interactions in a community context as well as their functional impacts on plant-associated microbial communities remains sparse. In this section, we discuss recent data obtained from microbial community profiling studies and associated ecological networks that underline the importance of microbe-microbe interactions for shaping microbial communities on plant tissues.

### The mycosphere: a niche hosting specific inhabitants

As part of the mycosphere, fungal hyphae or fruiting bodies have been recognized for a long time as important niches that can be colonized, both externally and internally, by specific bacterial taxa, including *Pseudomonas* strains and bacteria from the *Oxalobacteraceae*, *Bacillaceae*, and *Burkholderiaceae* families, among others [188–192]. Fungal exudates seem to play a specific role for mycosphere colonization by stimulating the growth of specific bacteria or inducing changes in bacterial community structure [193–195]. Particularly, exudates produced by the arbuscular mycorrhizal fungus *Rhizophagus irregularis* have been shown to stimulate bacterial growth and modify bacterial community structure, which is marked by an increased abundance of several Gammaproteobacteria [194]. Notably, bacterial ability to colonize the mycosphere correlates with their ability to use particular carbonaceous compounds abundantly found in mycosphere exudates such as l-arabinose, l-leucine, m-inositol, m-arabitol, d-mannitol, and d-trehalose [195]. Analysis of the soil bacterial community in the presence and absence of the arbuscular mycorrhizal fungus *Glomus hoi* using a microcosm experiment also revealed the significant effect of the fungus on bacterial community structure and suggests that nitrogen export by the fungus is an important driving force explaining bacterial community shift [196].

Recent studies have analyzed the bacterial diversity associated with mycorrhizal root tips, revealing the complexity of the interactions between mycorrhizal fungi and their associated bacterial microbiota in the mycorrhizosphere [134, 197–200]. Specifically, some bacterial orders (Burkholderiales and Rhizobiales) were reproducibly found within ectomycorrhizal root tips, indicative of a tight fungal-bacterial association [197]. Using microcosm experiments and germ-free *Pinus sylvestris*, Marupakula and collaborators recently found that root tips colonized by three different ectomycorrhizal fungi host statistically distinct bacterial communities. Although all three mycorrhizal types tightly associate with high abundance of *Burkholderia*, specific bacterial signatures could be detected for each fungus [200]. Similar to the mechanisms described for the mycorrhizosphere [201], it is therefore likely that numerous plant-associated fungi could indirectly impact bacterial communities by different means such as changes in nutrient availability, modulation of environmental pH, production of fungal exudates, or nutrient competition.

### Influence of pathogen invasion on the plant microbiota

Plant infection by pathogenic microbes often correlates with microbial community shifts in different plant compartments, including seeds [202], roots [203], wood [204], and leaves [87]. Analysis of the impact of two microbial invaders, the bacterial strain *Xanthomonas campestris* pv. *campestris* (*Xcc*) 8004 and the fungal isolate *Alternaria brassicicola* (*Ab*) Abra43 on the structure of seed-associated microbial assemblages in *Raphanus sativus*, indicates the different effect on the endogenous seed microbiota. The bacterial strain *Xcc* 8004 has no effect on microbial assemblages, whereas seed invasion by the fungal pathogen massively perturbs the resident fungal seed microbiota. Seed invasion by the pathogenic fungus explains ~ 60% of the variation of fungal communities observed between infected and non-infected seeds, likely due to fungal-fungal competition for resources and space [202]. Infection of oak leaves by the obligate filamentous pathogens *Erysiphe alphitoides* (powdery mildew fungus) or *A. thaliana* leaves by *Albugo* sp. (oomycete) is accompanied by significant changes in the composition of the phyllosphere microbiota [87, 205]. Notably, the pathogen *Albugo* has strong effects on epiphytic and endophytic bacterial colonization by decreasing species richness and stabilizing the community structure, which has been validated by manipulation experiments under controlled laboratory conditions [87]. Based on microbial correlation networks, Jakuschkin and collaborators identified 13 bacterial and fungal OTUs that significantly associate, either negatively or positively, with powdery mildew disease. Although the protective activities conferred by the corresponding microbes have not been validated yet, a direct antagonistic effect of *Mycosphaerella punctiformis* on *E. alphitoides* has been suggested [205]. Significant associations were also found between the composition of the endogenous fungal microbiota in poplar leaves and rust symptom severity, suggesting that resident foliar fungal endophytes can enhance or attenuate disease severity in wild trees [206]. Taken together, these data indicate a tight link between pathogen invasion and the microbial community structure *in planta* that likely results from the combined effect of microbe-microbe and microbe-host interactions.

### Co-occurrence and co-exclusion relationships among plant microbiota members

Until recently, microbial profiling data were primarily used to characterize the overall structure of plant-associated microbial communities as well as to determine the contribution of different factors on community structure. Currently, bioinformatics tools have been also developed to infer microbial co-occurrence networks from community profiling or metagenomic data [207, 208]. Microbial association networks, built based on pairwise comparisons between abundance profiles of individual taxa, allow the identification of possible connections (either positive, neutral, or negative) among plant microbiota members [32, 41, 87, 209]. Although these correlations do not necessarily predict causal relationships [210], analysis of plant-associated microbial networks tends to indicate that positive correlations dominate among microbes from the same kingdom, whereas negative interactions primarily occur through inter-kingdom microbe-microbe interactions [87]. These results suggest that evolutionary selection might have primarily favored competitive mechanisms between phylogenetically distant microbial groups, rather than between closely related taxa. Nonetheless, intra-kingdom competition through antibiotic secretion is known to sculpt bacterial networks in the rhizosphere, since the pattern of co-association was found to correlate with *Streptomyces* antagonistic activity [187]. Furthermore, it has been recently shown that microbial network analysis allows the identification of microbial taxa that positively associate with the absence of root infection by *Rhizoctonia solani* [211] or leaf infection by *E. alphitoides* [205]. These microbial taxa represent putative biocontrol agents that can be further validated through antagonistic activity tests and may represent interesting candidates for disease management. These sparse examples illustrate the power of microbial networks for identifying putative functional links among microbiota members. It remains nonetheless crucial to subject microbial network architecture to experimental testing in order to enable the transition from correlation- to causation-based studies.

### Microbial hubs as modulators of plant-associated microbial communities

Microbial network analysis represents an elegant way to identify specific microbes that have a more central position in the network, often defined as “keystone” species or “hubs.” These microbes frequently co-occur with other taxa (highly connected to other microbes within the network) and likely exert a strong influence on the structure of microbial communities (Fig. 3) [87, 208]. A comprehensive survey of bacterial, fungal, and oomycetal communities associated with the leaves of *A. thaliana* revealed the presence of few microbial hubs, such as the obligate biotrophic oomycete pathogen *Albugo* sp. and the basidiomycete yeast fungus *Dioszegia* sp., that act by suppressing the growth and diversity of other microbes. Other candidate bacterial hubs (*Comamonadaceae*) were also found to positively control the abundance of numerous phyllosphere bacteria [87]. Specific leaf-associated Cercomonads (Protists: Rhizaria: Cercozoa) were also recently shown to exert a significant effect on bacterial community composition. A less complex bacterial correlation network with a higher proportion of positive correlations was observed in the presence of protists, underlining the importance of predator-prey interactions for bacterial community structure [184]. In plant roots, Niu and colleagues have recently employed a simplified seven-species synthetic community that is representative of the maize root microbiota to study the role of *in planta* interspecies interactions in altering the host health and the establishment of root-associated bacterial communities [212]. Notably, the removal of one community member, *Enterobacter cloaceae*, caused a significant reduction in species richness indicating that *E. cloaceae* plays the role of “keystone” species within the seven-species community. In perennial plants, network analysis of mycorrhizal and endophytic fungi from beech trees (*Fagus* sp.) revealed the presence of two distinct microbial networks, consisting of diverse functional groups of mycorrhizal and endophytic fungi. Importantly, a different fungal hub dominates in each module (either *Oidiodendron* sp. or *Cenococcum* sp.), suggesting that diverse fungal hubs can differentially sculpt microbial assemblages within a single plant population [213]. However, microbial hub species identified through co-occurrence network analysis could represent generalist microbes that are reproducibly and abundantly found in plant tissues. These microbial hub species likely act on microbial communities either directly via microbe-microbe interactions and/or indirectly through (1) cascade modifications in the interconnected microbial network, (2) competition for space and nutrients, (3) alteration of the host immune system, or (4) modification of the host physiology. Validating the functional role of microbial hubs and determining the molecular mechanisms used by these microbes to modulate microbial community structure must now be prioritized using microbiota reconstitution experiments with germ-free plants.

## Cascading consequences of intermicrobial interactions on plant growth and health

Although competitive and cooperative interactions significantly impact plant-associated microbial assemblages, these microbial interactions might also alter plant growth and fitness in beneficial or deleterious ways. Although some correlations were observed between microbial community composition and plant host phylogeny [37, 39, 214], it is likely that a core plant microbiota has evolved with terrestrial plants (lycopods, ferns, gymnosperms, and angiosperms) over 450 million years [214] (Fig. 2). Therefore, it is plausible that these co-occurring core microbiota members have evolved, in parallel, niche-specific inter-microbial interactions strategies that impact plant growth and health.

### Intermicrobial interactions and plant growth promotion

Bacterial-mycorrhizal-plant relationships have been intensively studied since this microbial interplay can provide a direct benefit for the host plant [215]. The interaction between mycorrhizal fungi and specific rhizobacteria promotes the establishment and functioning of mycorrhizal symbioses with the plant host, including both endo- and ectomycorrhizal interactions [13, 216–220]. These so-called “helper” bacteria are able to act at several levels: (1) they increase the receptivity of the root to mycorrhizal fungi, (2) enhance soil conduciveness to the fungus, (3) promote germination of fungal spores, and (4) enhance mycelium survival [217]. Furthermore, this relationship appears to be specific, since some bacteria isolated from specific mycorrhizal fungi have antagonistic activities towards other phylogenetically unrelated fungi [217]. Beyond mycorrhiza helper bacteria, some bacterial endosymbionts of root-associated fungi also directly affect the plant host, as demonstrated for *Rhizobium radiobacter* F4. This *Serendipita indica*’s (formerly *Piriformospora indica*) endosymbiont is able to grow in the absence of its fungal host and can promote plant growth and resistance to plant leaf pathogens independently from *S. indica*, suggesting that *S. indica*-mediated plant growth promotion is partly mediated by its bacterial endosymbiont [221, 222] or by other bacterial members influencing fungal growth [223].

In nature, most land plants are co-colonized by fungal and bacterial symbionts, as well as a staggering diversity of endophytic and pathogenic microbes [59, 61]. However, it remains unclear how the competing demand of multiple partners is balanced in plant roots to maintain a beneficial output. A focus of interest is the cooperation between mycorrhizal fungi and nitrogen-fixing bacteria. These important members of the root microbiota are widespread and co-occur in the roots of many plant species [216]. Interestingly, it has been recently shown that these microbes can complement each other to maximize nutrient acquisition in the host and act synergistically to promote plant diversity and productivity [10]. Although the direct role of microbe-microbe interaction in this process is likely minor, mixed microbial consortia could, nonetheless, indirectly stimulate ecosystem functioning and plant productivity through different resource use strategies.

### Intermicrobial interactions and disease suppression

Soil bacterial communities from different taxonomic groups have an important biocontrol potential in the so-called “disease-suppressive” soils. In these soils, plants are less affected by pathogenic microbes due to the effect of their surrounding microbiota. Specifically, it has been proposed that fungal oxalic acid produced by the fungal root pathogen *Rhizoctonia solani* or compounds released from plant roots under attack promotes the growth of particular bacterial families (*Oxalobacteraceae* and *Burkholderiaceae*), leading to a bacterial community shift and the activation of bacterial stress and antagonistic responses that restrict the growth of the fungal pathogen [6, 224]. Furthermore, it has been shown that *Streptomyces* strains isolated from disease-suppressive soils can produce different VOCs with antifungal activity [225]. Other *Streptomyces* species have also been isolated from disease-suppressive soils from a strawberry field [226]. These bacteria have been found to produce an antifungal thiopeptide targeting fungal cell wall biosynthesis in *Fusarium oxysporum*, suggesting that different bacterial species use different competitive mechanisms [226].

Similarly, Santhanam and colleagues have elegantly demonstrated how root-associated bacteria provide an effective rescue to *Nicotiana attenuata* from the sudden-wilt disease. Seed inoculation with a core consortium of five bacterial isolates naturally adapted to the environment provides an efficient plant protection under field conditions, underlining the importance of using locally adapted microbiota members to control plant disease [227]. In the phyllosphere, it has been shown that the leaf surface microbiota, together with endogenous leaf cuticle mechanisms, leads to *A. thaliana* resistance against the broad host range necrotrophic fungal pathogen *Botrytis cinerea* [7]. Although it is not clear whether these bacterial communities were already stable or restructured after pathogen attack, it is likely that the plant actively recruits disease-suppressive bacteria during seed production or germination [19, 228]. Although many examples illustrate the biocontrol activity of plant-associated microbiota members, the molecular mechanisms leading to pathogen growth suppression on plant tissues remain sparse. Recently, it has been shown that the millet bacterial endophyte *Enterobacter* sp. can promote both growth and bending of millet root hairs, resulting in a multilayer root-hair endophyte stack that efficiently prevents entry by the fungal pathogen *Fusarium*. *Tn5*-mutagenesis further demonstrated that bacterial biocontrol activity requires c-di-GMP-dependent signaling, diverse fungicides, and resistance to a *Fusarium*-derived antibiotic [111].

Although it is known that the plant-associated microbiota can prevent disease, it remains difficult to engineer functionally reliable synthetic microbial consortia that promote plant growth and suppress disease. Reductionist approaches with synthetic microbial consortia suggest that pathogen suppression increases when the diversity of the bacterial consortium increases. It has been shown that complex *Pseudomonas* species consortia better protect tomato plants against the root pathogen *Ralstonia solanacearum* than low-complexity *Pseudomonas* spp. consortia, due to the combined action of antagonistic activities and resource competition [229]. Similarly, Wei and collaborators showed that disease incidence is reduced when the trophic network favor resource competition between non-pathogenic *R. solanacearum* and a pathogenic strain, due to overlap in resources acquisition [152]. These highlighted examples provide evidence that microbial diversity, resource competition, and intermicrobial antagonism are important factors to consider for engineering functionally relevant microbial consortia that efficiently suppress plant diseases.

### Intermicrobial interactions and disease facilitation

Intermicrobial interactions do not necessarily impact plant fitness in a positive way but can also be deleterious for the plant by enhancing disease. For instance, the bacterial plant pathogen *Clostridium puniceum* secretes clostrubins (antimicrobial polyketide) to compete against other microbial pathogens and survive in aerobic environments [230]. It has been also shown that toxin production by the bacterial endosymbiont of the plant-pathogenic fungus *Rhizopus* is required for successful fungal colonization of rice plants, indicating that fungal-bacterial symbioses can also promote disease [140]. Recently, high-throughput fungal profiling methods, combined with manipulative experiments, have shed new light on the ecological importance of fungal endophytes for rust disease modification in wild trees. Authors specifically showed that certain fungal endophytes in the poplar phyllosphere could reduce rust disease symptoms, whereas others promote susceptibility [206]. Taken together, these studies clearly show that intermicrobial interactions are complex and can also mediate disease facilitation.

## Conclusions

Plants live in intimate association with complex and diverse microbial communities. Next-generation sequencing has already enabled us to explore different microbial groups through the targeting of specific microbial loci or using environmental metagenomes. Nonetheless, a more holistic understanding is still needed to better understand the intermicrobial interactions within the microbiota of plants and to better define the functional relevance of the microbial networks for holobiont fitness [18, 231]. Prokaryotic and eukaryotic microbes have evolved a myriad of cooperative and competitive interaction mechanisms that shape and likely stabilize microbial assemblages on plant tissues. However, most of the data are derived from one-to-one interaction studies, and only few incorporate complex microbial communities in controlled laboratory conditions to reconstitute the plant microbiota and to understand the role of intermicrobial interactions. Such experiments will shed new light on the fundamental principles that govern the assembly of complex microbial communities and the maintenance of host-microbial homeostesis. Combining both empirical approaches [168–170, 232] and computationally inferred association networks [233–236] will be crucial to understand the ecology of microbial interactions during plant-microbiota establishment, to better predict assembly and stability of natural or synthetic microbial communities, and to better define the dynamics of microbial community establishment in time and in space. Finally, it is important to consider microbe-microbe interactions to accept or reject the hologenome theory, which postulates that selection can operate on horizontally acquired plant microbiota members. According to this concept, it is likely that microbes that tightly associate with plants also evolve community level microbe-microbe interaction strategies that allow them to persist within the plant holobiont. It is now crucial to determine whether the microbiota of plant shows high heritability, and to dissect whether community-level selection occurs between the host and the microbial community and between members of the microbial community.

## Abbreviations

AHLs: *N*-acyl-*l*-homoserine lactone
CF: Cytophaga
cm: Centimeter
e.g: Exampli gratia
etc: Et cetera
OTUs: Operational taxonomic units
sp.: Species
VOCs: Volatile organic compounds
